# A Comprehensive Analysis of KRT19 Combined with Immune Infiltration to Predict Breast Cancer Prognosis

**DOI:** 10.3390/genes13101838

**Published:** 2022-10-12

**Authors:** Lusi Mi, Nan Liang, Hui Sun

**Affiliations:** Division of Thyroid Surgery, The China-Japan Union Hospital of Jilin University, Jilin Provincial Key Laboratory of Surgical Translational Medicine, Jilin Provincial Precision Medicine Laboratory of Molecular Biology and Translational Medicine on Differentiated Thyroid Carcinoma, 126 Xiantai Street, Changchun 130033, China

**Keywords:** KRT19, breast cancer, methylation, phosphorylation, immune infiltration

## Abstract

To date, no study has been conducted to explore the mechanism of KRT19 and the correlation between the expression of KRT19 and immune infiltration in breast cancer (BRCA). TCGA, TIMER2.0, UALCAN, and other databases were used to analyze the expression, prognostic roles, epigenetic variants, and possible oncogenic mechanisms of KRT19 in BRCA. As a result, KRT19 showed higher expression compared with the normal tissues in BRCA. In addition, the epigenetic variation in KRT19, including gene alteration, mutation type and sites, DNA methylation, RNA modification, and phosphorylation, showed diversity in BRCA. Further mechanistic exploration suggested that the IL-17 signaling pathway and estrogen response might play essential roles in the regulation of KRT19. Moreover, KRT19 has different regulatory biological functions in BRCA. More importantly, the expression of KRT19 was closely related to immune infiltration and combining the two could effectively predict overall survival. Finally, a nomogram based on genes associated with cancer-immunity cycle signatures, which could predict progress free interval, was constructed and evaluated successfully. In conclusion, KRT19 may play a role in the occurrence and development of BRCA through the IL-17 signaling pathway. Meanwhile, KRT19 combined with immune infiltration can evaluate the prognosis of BRCA patients.

## 1. Introduction

Breast cancer (BRCA) is one of the most common malignancies among women in the vast majority of countries worldwide, and is currently the leading cause of cancer morbidity and mortality in women [1]. As shown in Figure S1, Keratin 19 (KRT19) is a protein-coding gene located on chromosome 17q21-1, which encodes the protein CYFRA21.1, a member of the keratin family [2]. CYFRA21.1 is located in the epithelial cytoskeleton, with high specificity, which can be detected in other body fluids such as blood. Serum levels are significantly elevated in patients with a variety of malignancies, especially in lung cancer [3]. At present, CYFRA21.1 is mostly used in combination with other serological tumor markers such as CA153 to diagnose early BRCA [4]. In recent years, immune infiltration has made progress in the diagnosis and treatment of tumors. Numerous studies have now demonstrated a link between immune infiltration and prognosis and response to therapy in several human cancer types, especially in colorectal cancer [5]. Based on this, the growing interest in the field of immunotherapy has prompted intensive research into the BRCA immune microenvironment [6]. However, it is unclear whether KRT19 is associated with the immune microenvironment in BRCA. Meanwhile, the molecular mechanism of KRT19 in BRCA is rarely reported in the literature. Therefore, in this study, the TCGA database and bioinformatics methods were used to initially explore the oncogenic mechanism of KRT19 and the association between KRT19 and the immune microenvironment in BRCA, hoping to provide a potential target for BRCA treatment in the future.

## 2. Materials and Methods

### 2.1. Gene Expression and Clinicopathological Character Analysis

The Cancer Genome Atlas (TCGA) database is a comprehensive and collaborative project that contains genomic, proteomic, transcriptomic, epigenomic, and clinical data. It molecularly characterizes over 20,000 primary cancers and matches normal samples spanning 33 cancer types [7]. GTEx was a data resource and tissue bank established to study the relationship between genetic variation and gene expression in multiple human tissues. This release includes genotype data from approximately 714 donors and approximately 11688 RNA-seq samples across 53 tissue sites and 2 cell lines [8] The University of Alabama Cancer database (UALCAN) (http://ualcan.path.uab.edu/, accessed on 29 October 2021) was used to analyze the cancer OMICS data, providing protein expression analysis data from the Clinical Proteomic Tumor Analysis Consortium (CPTAC) [9,10]. The Human Protein Atlas (HPA, https://www.proteinatlas.org/, accessed on 29 October 2021) is a project aiming to consolidate all the human proteins in cells, tissues, and organs by various omics technologies, including antibody-based imaging, mass spectrometry-based proteomics, transcriptomics, and systems biology [11]. In the TCGA database, there were 1092 breast cancer tissues and 113 normal breast tissues included. In order to increase the reliability of the data comparison, 179 normal breast tissues were selected in the GTEx database as control group. In addition, to analyze the differential expression of KRT19 at the protein level in breast cancer, 125 breast cancer tissues and 18 normal breast tissues were included from the CPTAC database. The results were visualized by the UALCAN online tool.

### 2.2. The Genetic Alterations Analysis

We performed the genomic alterations analysis though the cBioPortal (http://www.cbioportal.org/, accessed on 28 October 2021), which is an online public database for the interactive exploration of cancer genomic datasets [12,13]. There were 1084 breast invasive carcinoma samples from the TCGA (PanCancer Atlas) database, selected to explore KRT19 alterations. MEXPRESS (https://mexpress.be/, accessed on 17 November 2021) is a tool designed for the easy visualization of TCGA expression, DNA methylation, clinicopathological characteristics, and the relationships between them [14]. Consequently, the analysis of DNA methylation was also performed via UALCAN and MEXPRESS. Moreover, UALCAN provided the protein phosphorylation information of BRCA. A statistical significance of *p* < 0.05 was considered for the protein phosphorylation analysis.

### 2.3. Enrichment Analysis of KRT19-Related Genes

The R package “DESeq2” was used to screen out DEGs between high and low KRT19 expression patients from 1092 BRCA samples of TCGA database. Genes with adjusted *p* value < 0.05 and |logFC| > 1 were considered statistically significant. Gene Ontology (GO) analyses and Kyoto Encyclopedia of Genes and Genomes (KEGG) pathways were performed for DEGs using the R package “GOplot” (version 3.3.3). Gene Set Enrichment Analysis (GSEA) was performed to distinguish hallmark pathways involved in the gene signature. The GSEA (version 3.0) was used and “h.all.v7.4.symbols.gmt” was set as the reference database. Pathways with a normalized *p* value < 0.05 and false-discovery rate (FDR) q value < 0.25 were considered as statistically significant. The top enriched pathways were selected by ranking the normalized enrichment scores (NESs). Sangerbox (http://www.sangerbox.com/tool, accessed on 27 July 2022) was used to accomplish GESA analysis [15].

### 2.4. Single-Cell Analysis

CancerSEA (http://biocc.hrbmu.edu.cn/CancerSEA/home.jsp, accessed on 17 November 2021) is the first dedicated database that aims to comprehensively decode the functional states of cancer cells at a single-cell resolution [16]. The 369 cells were used to explore the function of KRT19 expression. They were from the dataset “Braune EB. Stem Cell Reports, 2016 (PDX)”, which denotes patient-derived xenograft breast cancer samples.

### 2.5. Immunity Analysis

TISIDB (http://cis.hku.hk/TISIDB/index.php, accessed on 28 October 2021) is a public database for tumor and immune system interactions, which could integrate multiple heterogeneous data types. In this study, it was used to investigate correlations between KRT19 and immunoinhibitors [17]. TIMER2.0 (http://timer.cistrome.org/, accessed on 6 July 2022) is a comprehensive resource for the systematical analysis of immune infiltrates across diverse cancer types and it is widely used to explore tumor immunological, clinical and genomic characteristics [18].The Tumor Immunophenotype (TIP) database (http://biocc.hrbmu.edu.cn/TIP, accessed on 28 July 2022) is an online resource that can assess the immune microenvironment based on the cancer-immunity cycle [19]. From the TIP, 178 signature genes were collected, which engaged in the seven stages of the cancer-immunity cycle, including checkpoints, cytotoxic factors, chemokines, and major histocompatibility complex (MHC) molecules.

### 2.6. Construction and Evaluation of Nomogram

The “rms” program was used to create a predicted nomogram. In the nomogram scoring system, each variable was assigned a score, and the total score was calculated by adding the scores from all factors in each sample. To determine the consistency of the nomogram prediction and clinical observation in 1, 3 and 5-year progress free interval (PFI), calibration curves were utilized. The nomogram was evaluated using ROC curves for 1, 3 and 5-year survival. Meanwhile, the concordance index (C-index) was computed to determine the nomogram’s predictive potential.

## 3. Results

### 3.1. Expression of the KRT19 in BRCA and Clinicopathological Characteristics in BRCA Patients

**Figure 1:**
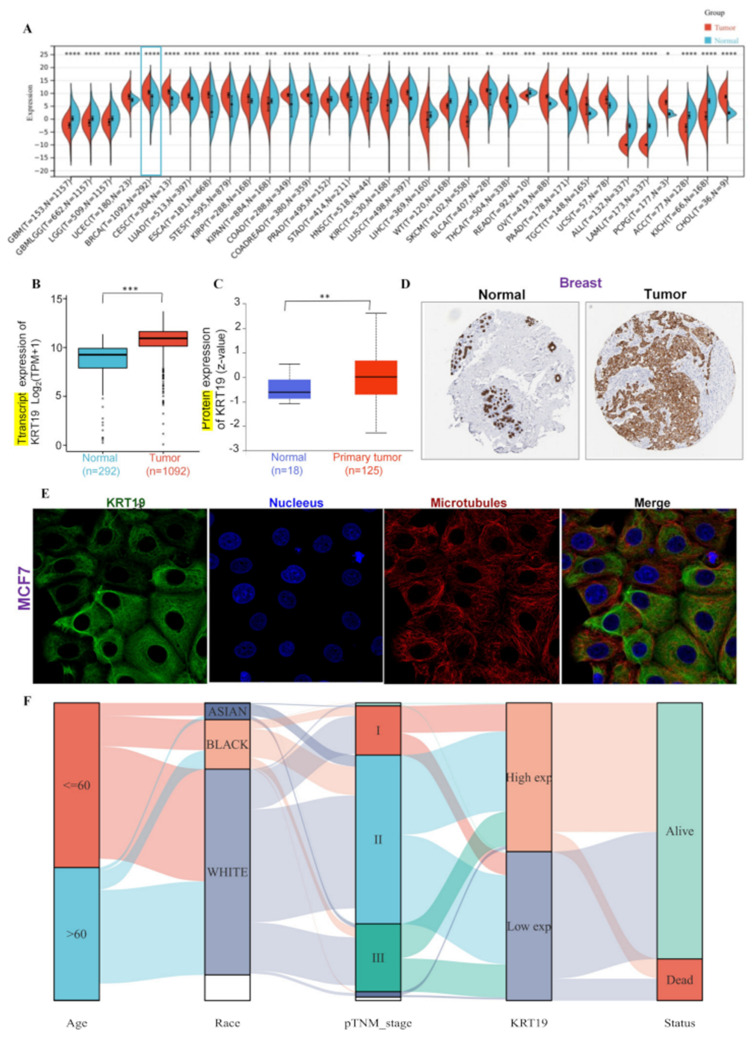
Differential expression of KRT19 in BRCA. (**A**). The expression status of the KRT19 gene in different cancers or specific cancer subtypes from TCGA and GTEx. * *p* < 0.05; ** *p* < 0.01; *** *p* < 0.001; **** *p* < 0.0001. (**B**). The expression of KRT19 in BRCA and breast normal tissues from TCGA and GTEx. (**C**). The expression level of KRT19 total protein between normal tissue and primary tissue of BRCA (UALCAN). (**D**). Immunohistochemical results of KRT19 in BRCA (HPA). (**E**). Immunofluorescence results of KRT19 in BRCA (HPA). (**F**). Correlation of KRT19 expression with clinicopathological characteristics of BRCA patients from TCGA. Each row represents a feature variable, different color represents different typing or stage, lines represent the distribution of the same sample in different feature variables.

Firstly, the difference between KRT19 expression in different cancer types and normal tissues was observed using the TCGA and GTEx database. As shown in Figure 1A, the vast majority of tumors showed higher expression of KRT19 at the mRNA level, especially significant in BRCA, KICH, and THCA etc. In BRCA, KRT19 still showed higher expression compared with the normal tissues (Figure 1B). The protein level of KRT19 in cancers was also explored from the CPTAC dataset. Compared to that in the breast normal tissues, KRT19 showed higher expression in BRCA (Figure 1C and Figure S2A). As shown in Figure 1D, the immunohistochemical images of KRT19 also suggested higher expression in BRCA. Meanwhile, the intracellular localization of KRT19 was observed from the perspective of immunofluorescence. It was indicated that in the MCF7 cell lines of BRCA, KRT19 was mainly localized on the microtubules (Figure 1E). In addition, we investigated the clinicopathological characteristics of BRCA patients with differential KRT19 expression. To explore the correlation between KRT19 expression and clinicopathological characteristics of breast cancer patients, we divided breast cancer patients into a KRT19 high-expression group and a low-expression group according to KRT19 expression. In total, 542 patients were included in the KRT19 high-expression group, and 541 patients were included in the KRT19 low-expression group. As shown in Table S1, the expression of KRT19 was significantly correlated with histological type, ER status, PR status and PAM50. In addition, we drew a sankey diagram to show the distribution trend of KRT19 expression in breast cancer patients with clinical characteristics. Each row represents a feature variable, a different color represents different typing or stage and lines represent the distribution of the same sample in different feature variables (Figure 1F). It was exciting that the AUC was as high as 0.852, suggesting that KRT19 might be used as a tumor marker for the diagnosis of BRCA (Figure S2B).

### 3.2. Epigenetic Variations and Genomic Heterogeneity of KRT19 in BRCA

**Figure 2:**
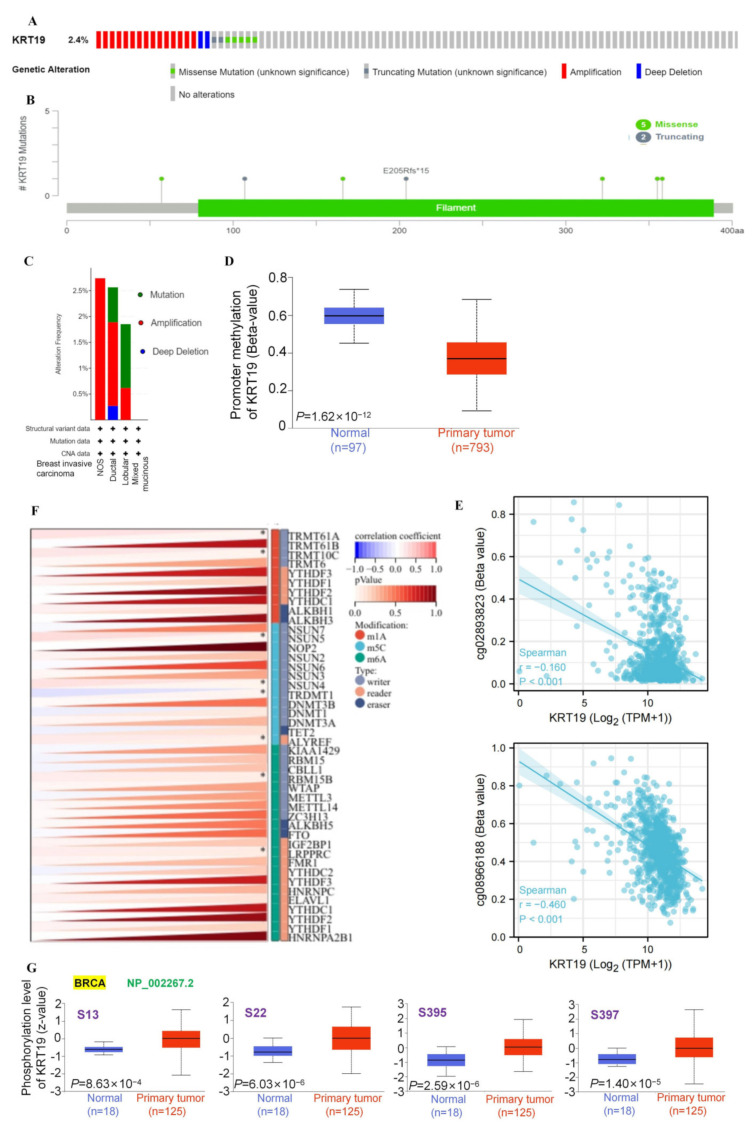
Epigenetic variations in KRT19 in BRCA. (**A**,**C**). The alteration frequency and the mutation type of KRT19 in BRCA (cBioPortal). (**B**). The mutation site. (**D**). The results of KRT19 promoter methylation levels in BRCA (TCGA). (**E**). The results of KRT19 methylation levels in BRCA by the Methylation450 platform. (**F**). RNA modification of KRT19 in BRCA. (**G**). Phosphorylation of KRT19 in BRCA (UALCAN).

To explore the potential functions of epigenetic variation in KRT19, we focused on its genetic alteration, DNA methylation, RNA modification, and phosphorylation in BRCA. Firstly, we observed the genetic alterations of KRT19 in BRCA samples of the TCGA cohorts. As shown in Figure 2A,C, in BRCA, amplication was the predominant form of genetic alteration in KRT19. And the frequency of amplication was the highest in Breast Invasive Carcinoma (NOS) (>2.5%). The types, sites, and case numbers of KRT19 genetic alterations are displayed in Figure 2B. Missense mutations were mainly concentrated in the filament domain, with a total of 4 loci. At the same time, truncating also occurred in the filament domain, including E205Rfs*15.

KRT19 methylation was investigated next. As shown in Figure 2E, compared to the breast normal tissues, promoter methylation levels of KRT19 were significantly downregulated in BRCA. Moreover, expression of KRT19 was negatively correlated with both cg02893823 and cg08966188 by the Methylation450 platform (Figure 2E). Figure S3A exhibited KRT19 copy numbers related to the CpG island in BRCA. Then, the RNA modification of KRT19 was explored. As shown in Figure 2F, KRT19 mainly underwent m5C modification, where NSUN5, NSUN4 and TRDMT1 acted as writers to catalyze the m5C modification of KRT19. TRMT61A and TRMT10C could promote the m1A change in KRT19. It was obvious that methyltransferase played an important role in the RNA modification of KRT19.

The expression levels of KRT19 phosphorylation between breast normal tissues and BRCA tissues were compared based on the CPTAC database. Figure 2G summarized the KRT19 phosphorylation sites and differences in BRCA, focusing on NP_002267.2 fragments. It was evident that the expression of KRT19 was upregulated at all the sites compared to normal tissue (S13, S22, S395, and S397). The potential functions and molecular mechanisms need to be explored further. Furthermore, we explored the relationship between KRT19 expression and genomic heterogeneity. KRT19 expression was negatively correlated with Tumor Mutational Burden (TMB). Unfortunately, we did not find a correlation between KRT19 expression and Micro Satellite Instability (MSI), Mutant-allele tumor heterogeneity (MATH) and Neoantigens (NEO) (Figure S3E).

### 3.3. Functional Annotation of KRT19-Associated DEGs in BRCA

**Figure 3:**
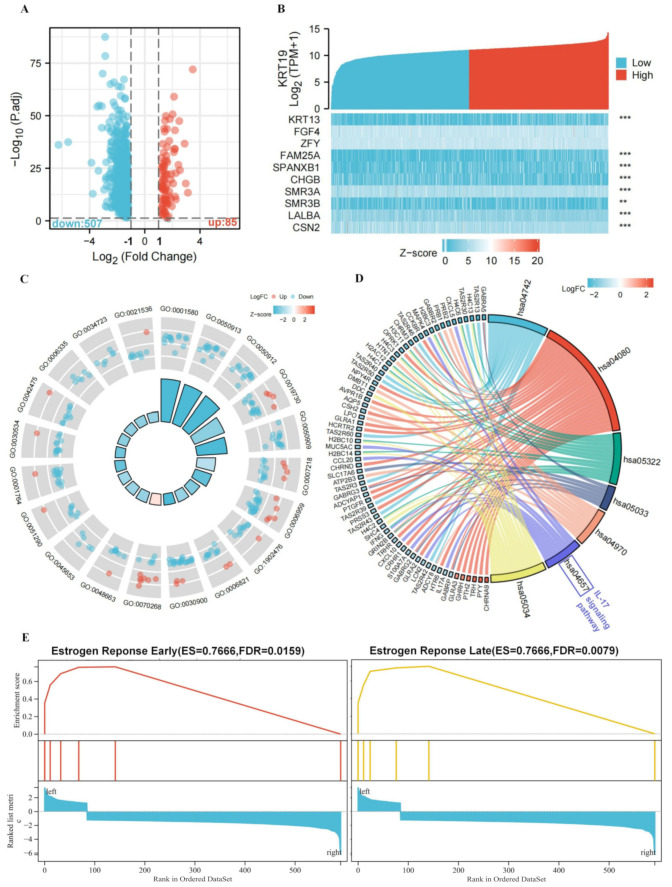
Functional annotation of differentially expressed genes (DEGs) with distinct KRT19 expression in BRCA. (**A**). DEGs associated with KRT19 in BRCA. Red dots represent upregulated 85 genes; blue dots represent downregulated 507 genes (**B**). Heatmap of the correlation between representative DEGs and KRT19. KRT13, FAM25A, SPANXB1, CHGB, SMR3A, SMR3B, LALBA, and CSN2 were significantly correlated with KRT19. ** *p* < 0.01, *** *p* < 0.001. (**C**). Biological process results of KRT19-associated DEGs. Red dots represent upregulated genes and blue dots represent downregulated genes. Blue rectangles suggest a negative correlation, and red rectangles the opposite. (**D**). KEGG pathway results of KRT19-associated DEGs. (**E**). The GESA results suggest that KRT19-related DEGs were enriched in the early estrogen response and late estrogen response pathways.

To evaluate the function of KRT19-associated DEGs in BRCA patients, BRCA patients were first divided into two groups according to the degree of KRT19 expression, which were high and low KRT19 expression, and searched for genes differentially expressed between these two groups. As shown in the volcano plot in Figure 3A, a fold change of 1.0 was selected as the boundary, and a total of 692 DEGs were screened, of which 85 were upregulated and 507 were included in downregulation. Representative DEGs were also illustrated by heatmaps (Figure 3B and Table S2). GO enrichment and KEGG pathway analysis were performed based on these DEGs next. As shown in Figure 3C,D and Figure S4, the several KRT19-related pathways were enriched, including IL-17 signaling pathway, neuroactive ligand-receptor interaction, and salivary secretion, etc. Surprisingly, early estrogen response and late estrogen response were found through GSEA enrichment analysis (Figure 3E). It might mean that KRT19 regulated the occurrence and development of BRCA by affecting the estrogen signaling pathway. This phenomenon deserves further investigation.

### 3.4. Biological Functions of the KRT19 in BRCA

**Figure 4:**
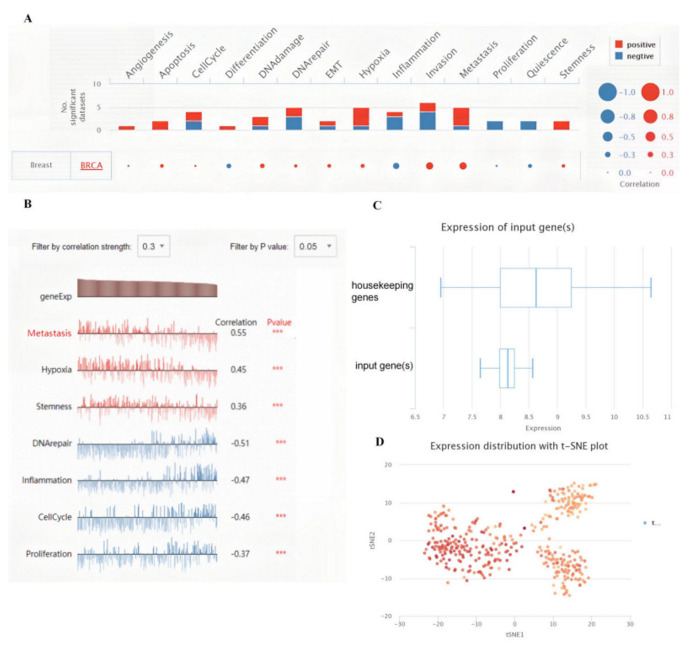
Biological functions of the KRT19 in BRCA from CancerSEA. (**A**,**B**). Single-cell analysis indicated that KRT19 positively regulates the metastasis, hypoxia, and stemness of BRCA cells and negatively regulates the DNA repair, cell cycle, proliferation, and inflammation of BRCA cells. *** *p* < 0.001 (**C**). The expression of KRT19 was slightly weaker compared to the housekeeping gene (**D**). The expression distribution of KRT19 by the t-SNE method.

In recent years, single-cell RNA sequencing (scRNA-seq) technology has emerged. It has coincided with transformative new methods to profile genetic, epigenetic, spatial, proteomic and lineage information in individual cells [20]. To investigate the biological functions of KRT19 in BRCA, single-cell analysis was performed using CancerSEA. The results indicated that KRT19 positively regulated the metastasis, hypoxia, and stemness of BRCA cells and negatively regulated the DNA repair, cell cycle, proliferation, and inflammation of BRCA cells (Figure 4A,B). We also further explored the expression distribution of KRT19. As shown in Figure 4C, the box diagram indicates that the expression of KRT19 was slightly weaker compared to the housekeeping genes and the stronger expression of KRT19 in the BRCA cells was observed by the t-SNE method (Figure 4D).

### 3.5. The Correlations between KRT19 Expression and Immunity in BRCA

**Figure 5:**
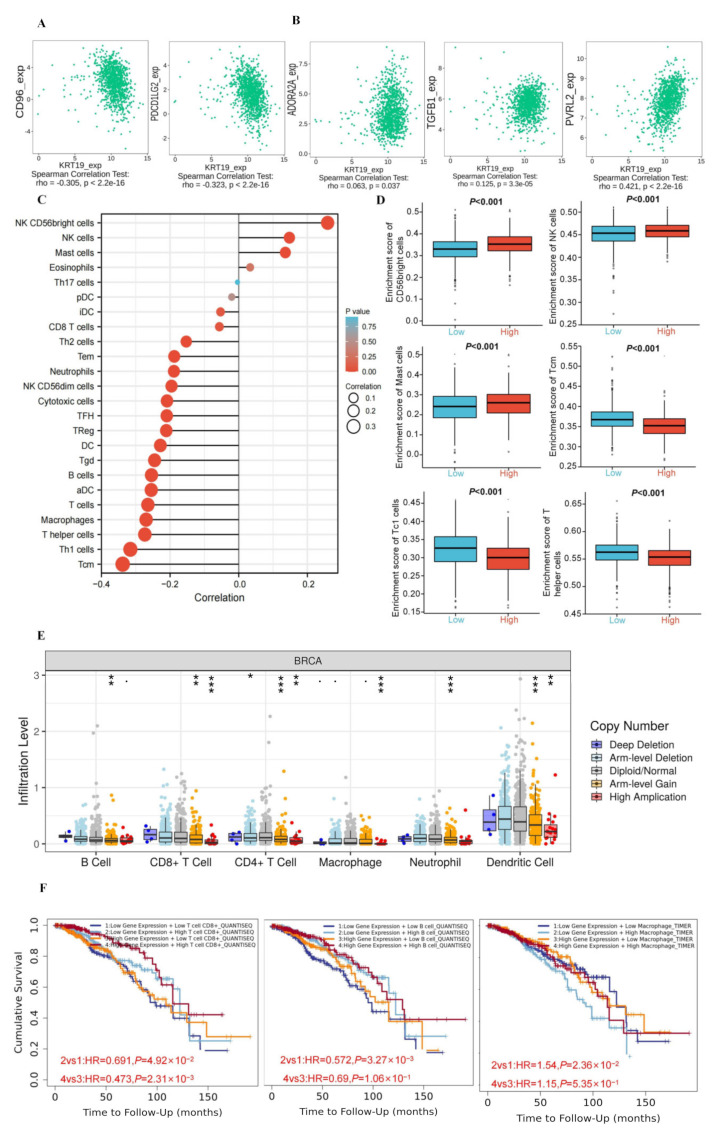
The correlations between KRT19 expression and immunity in BRCA. (**A**,**B**). The correlations of KRT19 expression and immunoinhibitors in BRCA (TISIDB). (**C**). The potential correlation between the expression level of the KRT19 and the infiltration level of immune cells in BRCA. (**D**). The expression of KRT19 were negatively correlated with the immune infiltration levels of Tcm, Th1 cells, and T help cells, positively correlated with the immune infiltration levels of NK CD56 bright cells, NK cells, and Mast cells. (**E**). Correlation of tumor infiltrating levels in BRCA and different somatic copy numbers’ alterations of KRT19. *p* < 0.1, * *p* < 0.05; ** *p* < 0.01; *** *p* < 0.001. (**F**). OS curves using combinations KRT19 expression and immune cells score (TIMER2.0).

In recent years, tumor immunotherapy has made great progress. Coincidentally, many DEGs were enriched in the IL-17 signaling pathway in the results of the KEGG pathway analysis. Therefore, it sparked our interest. Firstly, we assessed whether KRT19 expression was associated with immune checkpoint inhibitors by the TISIDB database. As a result, the remaining 22 immunoinhibitors were associated with KRT19 expression, except for KIR2DL1 and KIR2DL3. Among them, the majority of immunoinhibitors significantly negatively correlated with KRT19 expression, such as CD160. However, only ADORA2A, TGFB1, and PVRL2 showed slight correlations with KRT19 expression. This phenomenon deserves further in-depth investigation (Figure 5A,B, and Figure S5).

Moreover, the relationship between KRT19 expression and immune infiltration of BRCA tumor cells was analyzed. As shown in Figure 5C,D, the immune infiltration levels of the vast majority of immune cells were negatively correlated with the expression of KRT19, especially Tcm, Th1 cells, and T help cells. The immune infiltration levels of only three immune cells, including NK CD56 bright cells, NK cells, and Mast cells, were possibly correlated with the expression of KRT19. As shown in Figure 5E, the correlation between the somatic copy number alterations (SCNA) in KER19 and the level of immune infiltration of various immune cells was explored. For example, in Dendritic cells, we found the lowest level of immune infiltration with high amplication alterations in KRT19.

Furthermore, the Kaplan–Meier analysis was performed to detect the correlation between the aberrant KRT19 expression and immune cell infiltration with clinical performance. It was shown that in BRCA patients, taking OS as the end point of the study, when KRT19 was lowly expressed, low T cell CD8+ indicated poor prognosis compared with high T cell CD8+. However, low T cell CD8+ also indicated poor prognosis, while KRT19 was highly expressed. Similarly, combined KRT19 expression with B cell score analysis showed that BRCA patients with low expression of KRT19 and B cell presented the worse OS. Meanwhile, high Macrophage was found to suggest the shorter OS at the level of low expression of KRT19 (Figure 5F). It suggested that the combination of KRT19 and immune infiltration might become a new prognostic marker for BRCA.

### 3.6. Construction and Evaluation of Nomogram Based on Genes Associated with the Cancer-immunity Cycle Signatures

**Figure 6:**
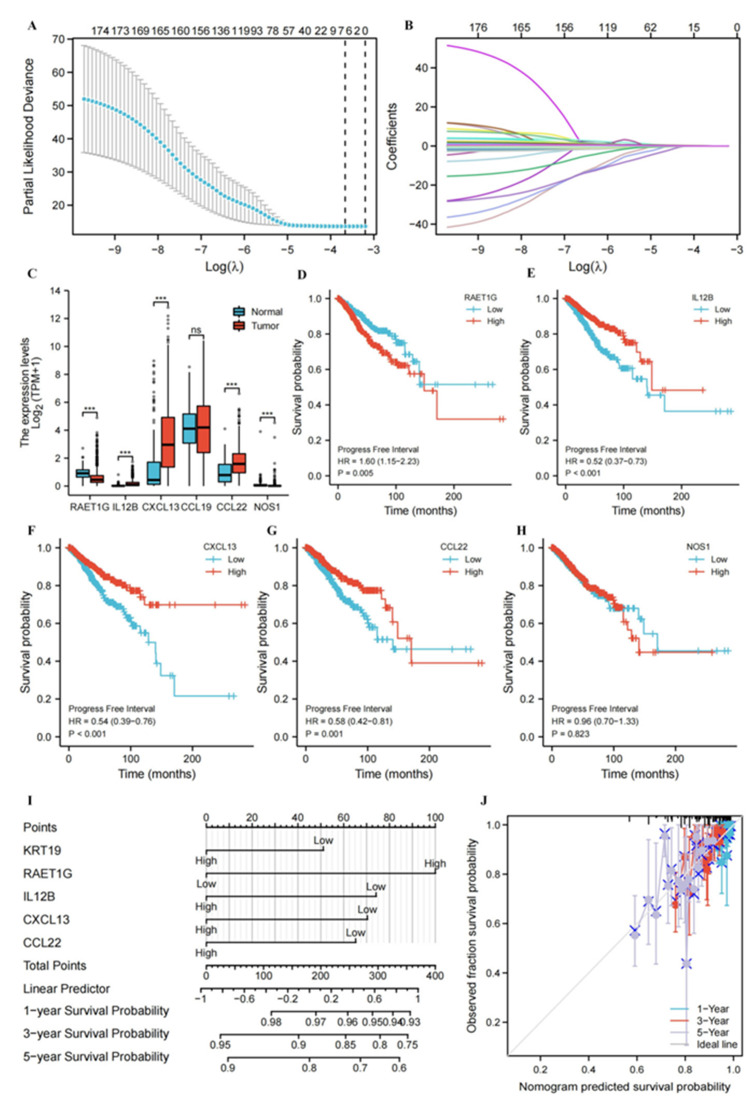
Construction and evaluation of nomogram based on genes associated with cancer-immunity cycle signatures. (**A**,**B**). Analysis of LASSO regression in TCGA database. The determination of “lambda” for optimal selection of gene signature. (**C**). The expression of RAET1G, IL12B, CXCL13, CCL19, CCL22, and NOS1 in BRCA and breast normal tissues. (**D**–**H**). The prognostic roles of RAET1G, IL12B, CXCL13, CCL22, and NOS1 in BRCA (PFI). (**I**). The nomogram is applied by adding up KRT19, RAET1G, IL12B, CXCL13, and CCL22. The total points projected on the bottom scales imply the probability 1, 3, 5 years PFI. (**J**). Calibration curves of the nomogram for the prediction of survival rates at 1, 3, 5 years, *** *p* < 0.001.

Given the above positive results of KRT19 and immune infiltration, the correlation between KRT19 and cancer-immunity cycle-related genes was further analyzed. As shown in Figure S5C, a Venn diagram of 178 cancer-immunity cycle-related genes in the TCGA cohort and 9 genes in the IL-17 signaling pathway was drawn. Additionally, common members of the two groups were CXCL10, CXCL5, CCL20, and IFNG. Therefore, LASSO analysis was performed on 178 cancer immune cycle-related genes to establish the best model for evaluating PFI in BRCA patients. The resultant change trajectory of each independent variable is depicted in Figure 6A, followed by the LASSO regression analysis. The confidence interval under each lambda is exhibited in Figure 6B. There were 6 cancer-immunity cycle-associated genes with an optimal λ value, including RAET1G, IL12B, CXCL13, CCL19, CCL22, and NOS1. Then, the difference between the expression of these 6 genes in BRCA and breast normal tissues was observed using the TCGA and GTEx database. As shown in Figure 6C, except for CCL19, RAET1G, IL12B, CXCL13, CCL22, and NOS1 were significantly highly expressed in breast cancer at the mRNA level. In addition, we paid special attention to the prognostic roles of RAET1G, IL12B, CXCL13, CCL22, and NOS1 in BRCA. Compared with the high expression group, when IL12B, CXCL13, and CCL22 were lowly expressed, the PFI of BRCA patients was shorter, suggesting poor prognosis. On the contrary, high expression of RAET1G indicated poor prognosis (Figure 6D–H). Table 1 displayed univariate and multivariate cox analyses of clinicopathological characteristics for PFI in BRCA. Finally, KRT19, RAET1G, IL12B, CXCL13, and CCL22 were included as prognostic factors in the nomogram, which had a C-index of 0.634 (Figure 6I). The calibration curve showed that the nomogram was reliable in predicting the possibility of 1, 3, 5 years PFI in BRCA (Figure 6J). These results demonstrated that the nomogram combining expression of KRT19, RAET1G, IL12B, CXCL13, and CCL22 had better predictive power for the PFI of BRCA patients, which might contribute to the efficacy assessment and managing patients.

## 4. Discussion

In this study, we analyzed the expression, epigenetic variants, the possible oncogenic mechanism and immunity of KRT19, providing a theoretical foundation for the possibility of KRT19 as a potential marker in BRCA.

It is well known that breast cancer is a common malignancy worldwide and a major cause of death in women. According to the latest data from the United States, the incidence of female breast cancer has been slowly increasing at a rate of about 0.5% per year since the mid-2000s [1]. KRT19, as a serum tumor marker for breast cancer, is mainly localized in the cytoskeleton and cytosol in cells (Figure S1B), which is currently more widely used in the early diagnosis of BRCA [4]. However, there are few studies on its role in the mechanism of BRCA. Therefore, based on previous studies, we used a series of bioinformatics approaches to explore in-depth the possible molecular mechanisms of KRT19 in BRCA in this article. Firstly, it was indicated that KRT19 was specifically highly expressed in breast cancer at the mRNA and protein levels. This is consistent with the study by Nuzhat N. Kabir et al. [21]. It is estimated that 5–10% of all breast cancer cases in women are associated with genetic susceptibility due to autosomal dominant gene mutations [22].

Therefore, we further explored the genetic alterations in KRT19 in breast cancer. We found that amplification was the main form of KRT19 alteration in breast cancer patients. DNA methylation is an epigenetic process where a methyl group is added to the cytosine base of the DNA, most commonly occurring at the CG dinucleotides, also known as CpG methylation. DNA methylation plays a vital role in cellular growth, differentiation, and disease pathogenesis. Aberrant DNA methylation is often considered a characteristic signature of cancer development [23,24]. In 2019, de Almeida et al. explored the relationship between DNA methylation and breast cancer, and identified new DNA methylation markers, including cg12374721 (PRAC2), cg18081940 (TDRD10) and cg04475027 (TMEM132C). It is promising as a diagnostic and prognostic marker for breast cancer and other cancer types [25]. In this study, we found that KRT19 methylation levels were significantly downregulated in breast cancer patients. Phosphorylation, one of the most widely studied post-translational modifications, regulates many cellular functions, including cell growth, differentiation, apoptosis, and cell signaling, in the healthy state. However, alterations in phosphorylation pathways can lead to serious disease consequences, especially cancers [26]. Therefore, the relationship between KRT19 expression and its phosphorylation in breast cancer patients was explored using the TCGA database. It was found that KRT19 expression was significantly upregulated at the sites, such as S13, S22, S395, and S397. It is suggested that KRT19 methylation and phosphorylation may play an important role in the occurrence and development of breast cancer, which needs further experiment to test and verify. In addition, there has been a significant amount of research on RNA modification in cancer in recent years. The dynamics of these RNA chemical modifications are orchestrated by coordinated actions of an array of writer, reader and eraser proteins. Deregulated expression of these RNA modifying proteins, including m6A, m1A, and m5C et al., can lead to many human diseases, including cancer [27]. In this study, we found that the expression of KRT19 was associated with many RNA-modified writer proteins. It suggested that KRT19 might affect the occurrence and development of BRCA via RNA modification. This interesting phenomenon needs further research and discussion.

Subsequently, we detected a total of 692 significant DEGs, of which 507 had upregulated gene expression and 85 had downregulated gene expression. The pathway enrichment analysis showed that 9 DEGs were enriched to the IL-17 signaling pathway, respectively. IL-17 is a highly versatile pro-inflammatory cytokine, crucial for a variety of processes, including host defense, tissue repair, the pathogenesis of inflammatory disease and the progression of cancer [28]. Currently, IL-17 has been shown to promote prostate adenocarcinoma while increasing matrix metalloproteinase 7 (MMP7) expression in mouse prostate [29]. The review by Alinejad V et al. comprehensively details the activity, signaling, and role of IL17B-IL17RB in BRCA [30]. Based on the results of this study, we suggest that the expression of KRT19 may influence breast cancer development by affecting the IL-17 signaling pathway. This hypothesis depends on further experimental validation.

To further evaluate the function of KRT19, we performed data analysis using CancerSEA. The results showed that KRT19 may influence the development of BRCA through metastasis and other pathways. In recent years, preliminary studies on immunotherapy have focused on targeting immune checkpoints as an effective strategy to enhance beneficial anti-tumor immune responses. PD-1 is considered as an immune checkpoint. Meanwhile, it is now also more relevantly studied to prevent autoimmune responses by inducing apoptosis of antigen-specific T cells and inhibiting apoptosis of regulatory T cells [31]. At the same time, the growing interest in the field of immunotherapy has promoted an in-depth study of the immune microenvironment of breast cancer. In this context, tumor-infiltrating lymphocytes have emerged as a clinically relevant and highly reproducible biomarker capable of influencing breast cancer prognosis and response to therapy [6]. According to our findings, KRT19 expression was negatively correlated with most immune inhibitors such as CD160, but positively correlated with the level of infiltration of immune cells such as NK cells. It has been shown that CD160 is a unique receptor for activated NK cells [32]. Thus, it is hypothesized that KRT19 expression promotes the development of BRCA by increasing the function of NK cells. Of course, this idea still needs further investigation and experimental validation. Based on the close relationship between the expression of KRT19 and immune infiltration, we made further exploration about cancer-immunity cycle. Le Bouteiller et al. believe that for an anticancer immune response to lead to the effective killing of cancer cells, a series of stepwise events must be initiated and allowed to proceed and expand iteratively. These steps were referred to as the cancer-immunity cycle [33]. Surprisingly, we screened out genes related to BRCA prognosis and successfully established a prognostic model that could predict PFI. In the future, we hope to collect clinical data to further validate this model.

There were several limitations in this study. Firstly, all the original data were from online public databases, not real-world samples. Next, many preliminary experiments on the mechanism of KRT19 require further experiments to verify. Lastly, as for the correlation between KRT19 and the clinicopathological characteristics within breast cancer patients, more clinical specimen data should be collected and verified.

## 5. Conclusions

KRT19 may play a role in the occurrence and development of BRCA through IL-17 signaling pathway. Meanwhile, KRT19 combined with immune infiltration can evaluate the prognosis of BRCA patients.

## Figures and Tables

**Table 1:** Univariate and multivariate Cox analyses of clinicopathological characters for PFI in BRCA.

Character	Total (N)	Univariate Analysis		Multivariate Analysis	
		Hazard Ratio (95% CI)	*p* Value	Hazard Ratio (95% CI)	*p* Value
Age	1082				
≤60	601	Reference			
>60	481	1.253 (0.904–1.738)	0.175		
Race	993				
Asian	60	Reference			
White	753	0.832 (0.338–2.049)	0.689		
Black or African American	180	0.947 (0.364–2.465)	0.912		
T stage	1079				
T1	276	Reference			
T2	629	1.615 (1.042–2.501)	0.032	1.342 (0.818–2.204)	0.244
T3	139	2.213 (1.290–3.798)	0.004	1.211 (0.615–2.385)	0.579
T4	35	6.258 (3.262–12.008)	<0.001	2.775 (1.227–6.279)	0.014
N stage	1063				
N0	514	Reference			
N1	357	1.981 (1.331–2.948)	<0.001	1.481 (0.957–2.290)	0.078
N2	116	2.481 (1.441–4.272)	0.001	2.154 (1.200–3.864)	0.010
N3	76	4.961 (2.833–8.688)	<0.001	2.436 (1.159–5.124)	0.019
M stage	922				
M0	902	Reference			
M1	20	8.315 (4.829–14.315)	<0.001	3.408 (1.699–6.838)	<0.001
KRT19	1082				
Low	540	Reference			
High	542	0.856 (0.617–1.187)	0.350		
RAET1G	1082				
Low	541	Reference			
High	541	1.604 (1.152–2.234)	0.005	1.912 (1.318–2.774)	<0.001
IL12B	1082				
Low	541	Reference			
High	541	0.519 (0.371–0.726)	<0.001	0.809 (0.511–1.279)	0.364
CXCL13	1082				
Low	540	Reference			
High	542	0.543 (0.389–0.758)	<0.001	0.756 (0.491–1.166)	0.206
CCL22	1082				
Low	540	Reference			
High	542	0.584 (0.420–0.812)	0.001	0.714 (0.472–1.079)	0.110

## Data Availability

The datasets used and/or analyzed during the current study are available from the corresponding author on reasonable request.

## Supplementary Materials

The following are available online at https://www.mdpi.com/article/10.3390/genes13101838/s1, Figure S1. The location and expression of KRT19 in normal cells and tissues. Figure S2. Protein expression and diagnostic efficacy of KRT19 and in BRCA. Figure S3. Epigenetic variations of KRT19 in BRCA. Figure S4. CC and MF of DEGs with distinct KRT19 expression in BRCA. Figure S5. The correlation of KRT19 expression and immuoinhibitors in BRCA. Table S1. Correlation between KRT19 expression and clinicopathological characteristics in BRCA patients. Table S2. Correlation between KRT19 and co-expression molecules. Table S3. The GO results of DEGs with distinct KRT19 expression in BRCA.
