# Supplementary material for: A Comprehensive Analysis of KRT19 Combined with Immune Infiltration to Predict Breast Cancer Prognosis
Source: Genes (Basel). 2022 Oct 12;13(10):1838. doi: 10.3390/genes13101838 (PMC9602083; doi:10.3390/genes13101838)
Supplement: Supplementary file 1 [file genes-13-01838-s001.zip › Table S1.pdf]

Table S1.Correlation between KRT19 expression and clinicopathological features in BRCA.

Characteristic	Low expression of KRT19	High expression of KRT19	***P***
n	541	542	
T stage, n (%)			0.291
T1	125 (11.6%)	152 (14.1%)	
T2	326 (30.2%)	303 (28.1%)	
T3	70 (6.5%)	69 (6.4%)	
T4	19 (1.8%)	16 (1.5%)	
N stage, n (%)			0.667
N0	267 (25.1%)	247 (23.2%)	
N1	171 (16.1%)	187 (17.6%)	
N2	57 (5.4%)	59 (5.5%)	
N3	39 (3.7%)	37 (3.5%)	
M stage, n (%)			0.387
M0	472 (51.2%)	430 (46.6%)	
M1	8 (0.9%)	12 (1.3%)	
Pathologic stage, n (%)			0.132
Stage I	81 (7.6%)	100 (9.4%)	
Stage II	325 (30.7%)	294 (27.7%)	
Stage III	119 (11.2%)	123 (11.6%)	
Stage IV	6 (0.6%)	12 (1.1%)	
Race, n (%)			**0.027**
Asian	32 (3.2%)	28 (2.8%)	
Black or African American	76 (7.6%)	105 (10.6%)	
White	399 (40.1%)	354 (35.6%)	
Age, n (%)			0.519

<=60	306 (28.3%)	295 (27.2%)	
>60	235 (21.7%)	247 (22.8%)	
Histological type, n (%)			**0.090**
Infiltrating Ductal Carcinoma	389 (39.8%)	383 (39.2%)	
Infiltrating Lobular Carcinoma	89 (9.1%)	116 (11.9%)	
PR status, n (%)			**< 0.001**
Negative	223 (21.6%)	119 (11.5%)	
Indeterminate	3 (0.3%)	1 (0.1%)	
Positive	286 (27.7%)	402 (38.9%)	
ER status, n (%)			**< 0.001**
Negative	177 (17.1%)	63 (6.1%)	
Indeterminate	1 (0.1%)	1 (0.1%)	
Positive	335 (32.4%)	458 (44.3%)	
HER2 status, n (%)			0.939
Negative	300 (41.3%)	258 (35.5%)	
Indeterminate	6 (0.8%)	6 (0.8%)	
Positive	86 (11.8%)	71 (9.8%)	
PAM50, n (%)			**< 0.001**
Normal	27 (2.5%)	13 (1.2%)	
LumA	230 (21.2%)	332 (30.7%)	
LumB	80 (7.4%)	124 (11.4%)	
Her2	53 (4.9%)	29 (2.7%)	
Basal	151 (13.9%)	44 (4.1%)	
Menopause status, n (%)			0.149
Pre	123 (12.7%)	106 (10.9%)	
Peri	25 (2.6%)	15 (1.5%)	
Post	345 (35.5%)	358 (36.8%)	

Anatomic neoplasm subdivisions, n (%)			0.975
Left	282 (26%)	281 (25.9%)	
Right	259 (23.9%)	261 (24.1%)	
radiation_therapy, n (%)			0.862
No	216 (21.9%)	218 (22.1%)	
Yes	271 (27.5%)	282 (28.6%)	
OS event, n (%)			0.424
Alive	460 (42.5%)	471 (43.5%)	
Dead	81 (7.5%)	71 (6.6%)	
DSS event, n (%)			0.351
Alive	483 (45.4%)	495 (46.6%)	
Dead	47 (4.4%)	38 (3.6%)	
PFI event, n (%)			0.152
Alive	459 (42.4%)	477 (44%)	
Dead	82 (7.6%)	65 (6%)	
Age, meidan (IQR)	58 (48, 67)	59 (49, 67)	0.360
