# Supplementary material for: A Comprehensive Analysis of KRT19 Combined with Immune Infiltration to Predict Breast Cancer Prognosis
Source: Genes (Basel). 2022 Oct 12;13(10):1838. doi: 10.3390/genes13101838 (PMC9602083; doi:10.3390/genes13101838)
Supplement: Supplementary file 1 [file genes-13-01838-s001.zip › Table S2.pdf]

Table S2.Correlation between KRT19 and co-expression molecules

Target molecule	Other molecules	Correlation coefficient (Pearson)	*P* (Pearson)	Correlation coefficient (Spearman)	*P* (Spearman)
KRT19	KRT13	0.140	<0.001	0.283	<0.001
KRT19	FGF4	0.013	0.666	-0.037	0.214
KRT19	ZFY	0.078	0.010	0.013	0.654
KRT19	FAM25A	0.267	<0.001	0.361	<0.001
KRT19	SPANXB1	0.100	<0.001	0.145	<0.001
KRT19	CHGB	-0.075	0.012	-0.116	<0.001
KRT19	SMR3A	-0.105	<0.001	-0.166	<0.001
KRT19	SMR3B	-0.102	<0.001	-0.094	0.002
KRT19	LALBA	-0.093	0.002	-0.176	<0.001
KRT19	CSN2	-0.082	0.007	-0.241	<0.001
