# Supplementary figures and images for: A Comprehensive Analysis of KRT19 Combined with Immune Infiltration to Predict Breast Cancer Prognosis
Source: Genes (Basel). 2022 Oct 12;13(10):1838. doi: 10.3390/genes13101838 (PMC9602083; doi:10.3390/genes13101838)

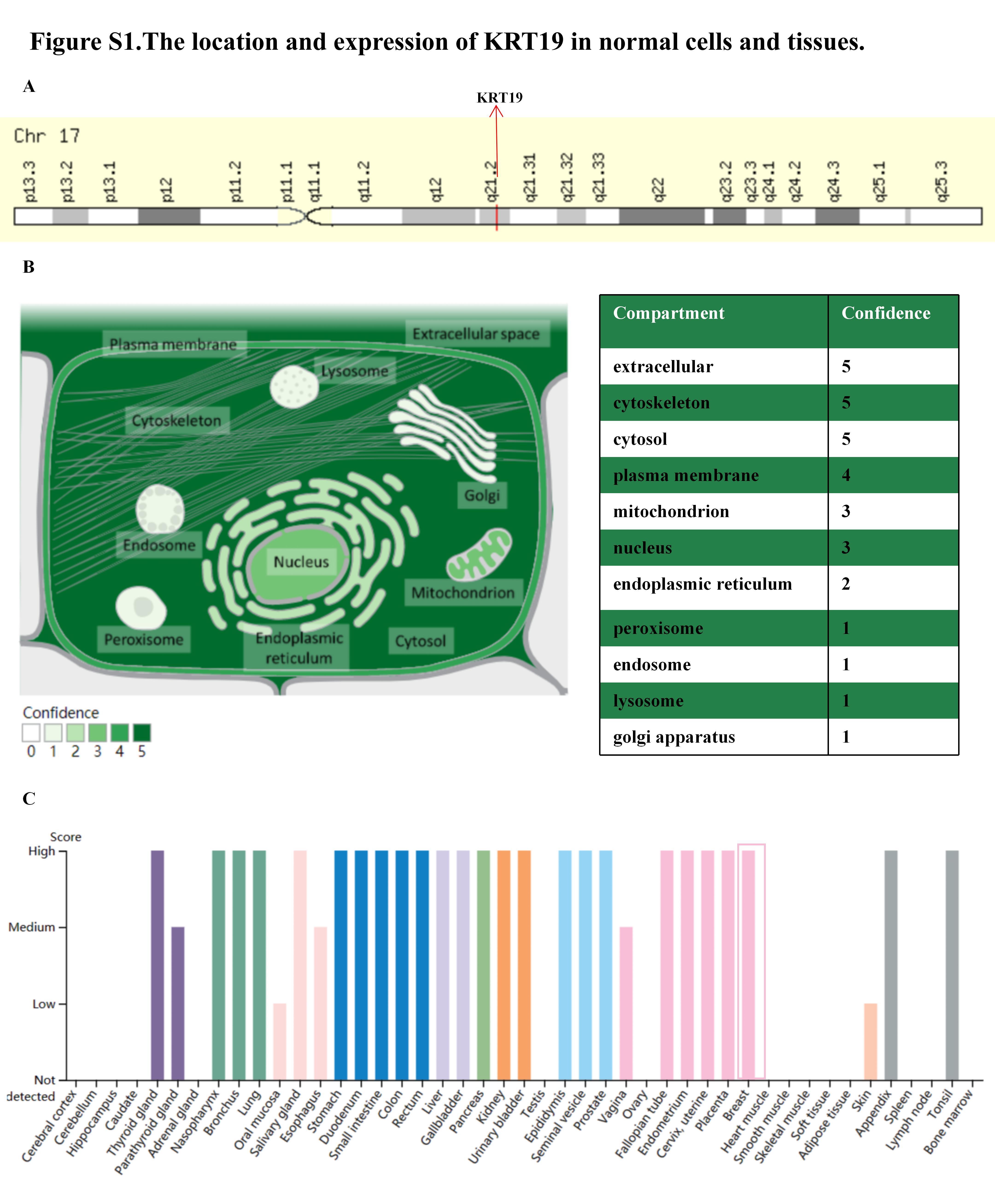

Supplement: Supplementary file 1 [file genes-13-01838-s001.zip › Figure S1.jpg]

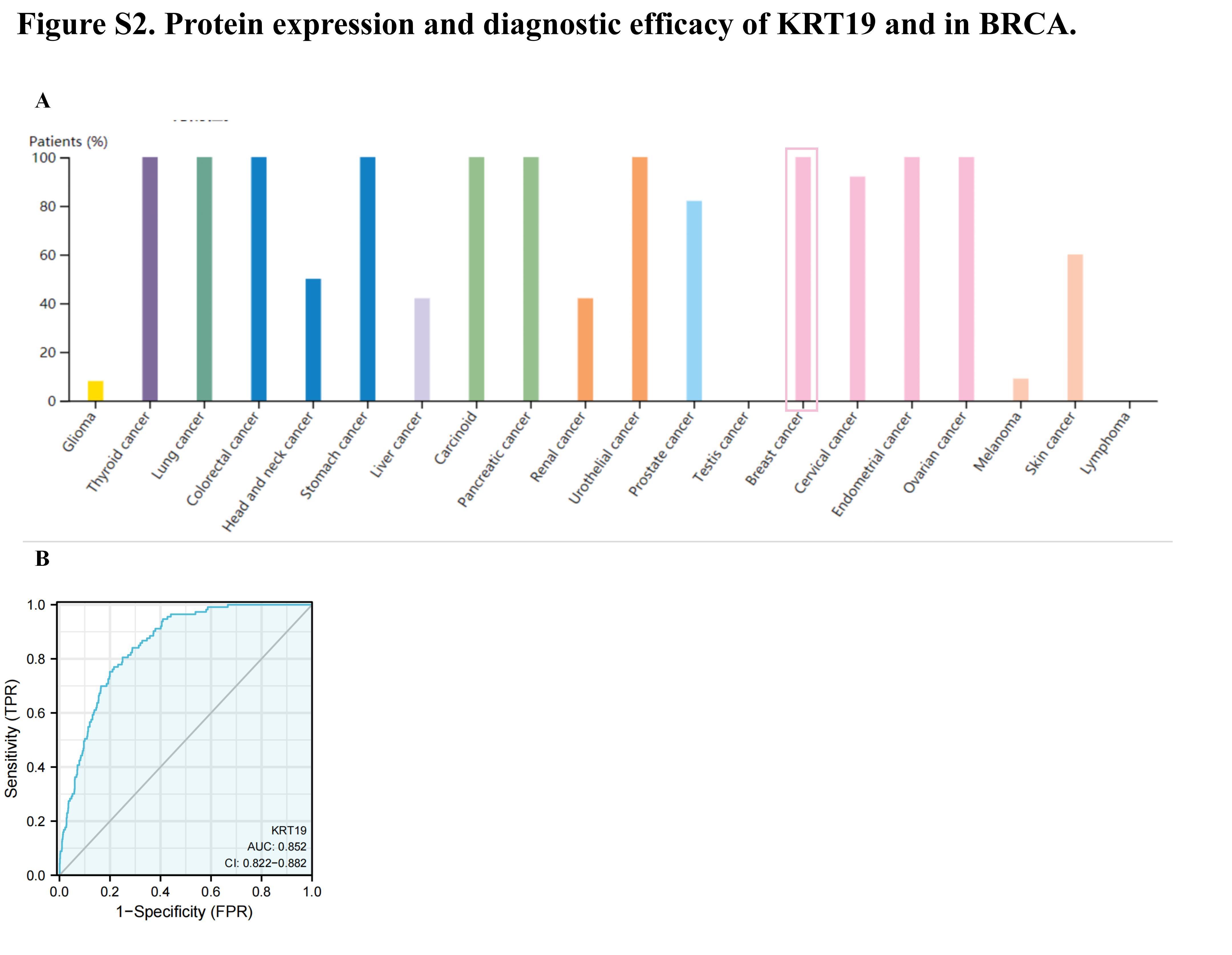

Supplement: Supplementary file 1 [file genes-13-01838-s001.zip › Figure S2.jpg]

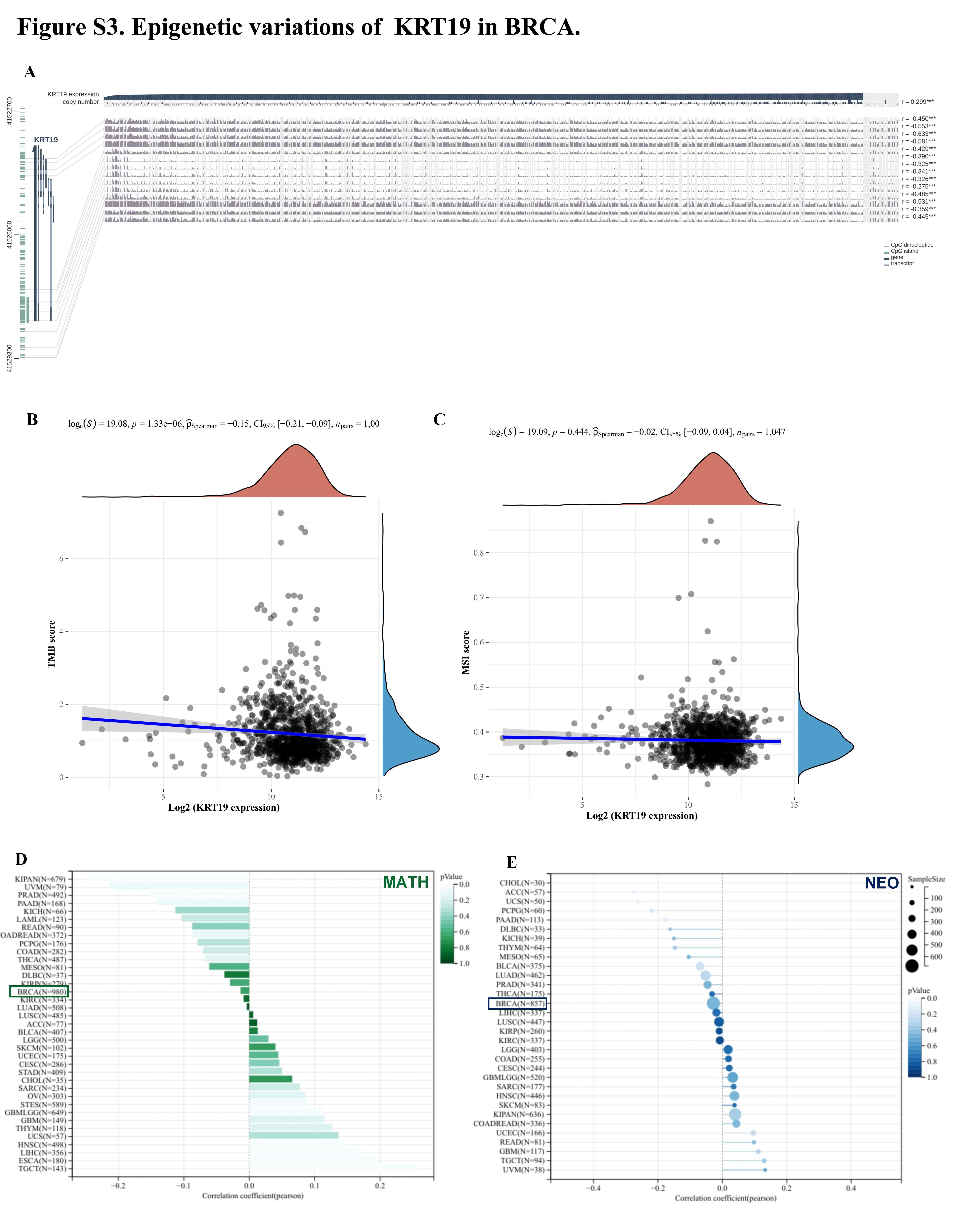

Supplement: Supplementary file 1 [file genes-13-01838-s001.zip › Figure S3.jpg]

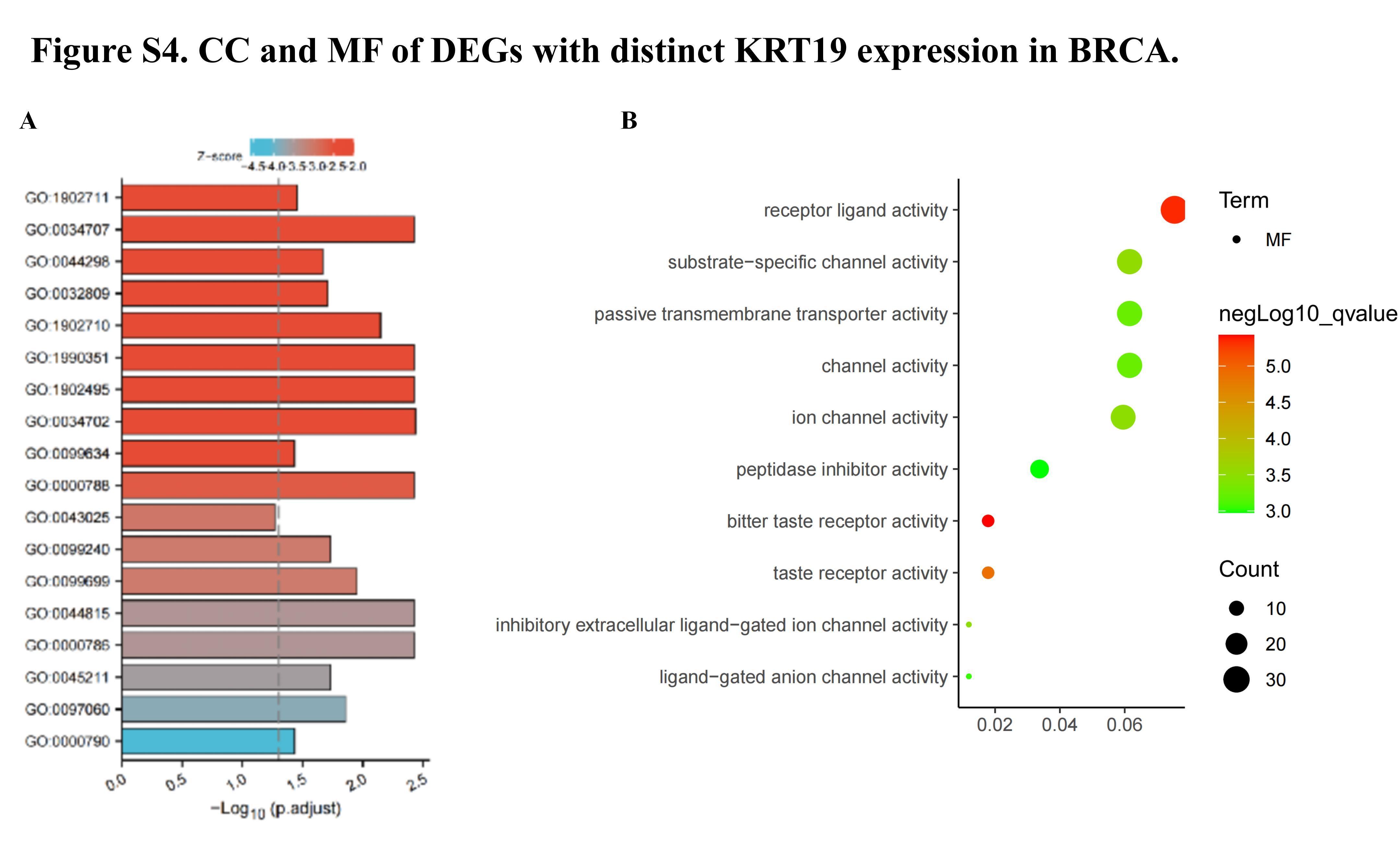

Supplement: Supplementary file 1 [file genes-13-01838-s001.zip › Figure S4.jpg]

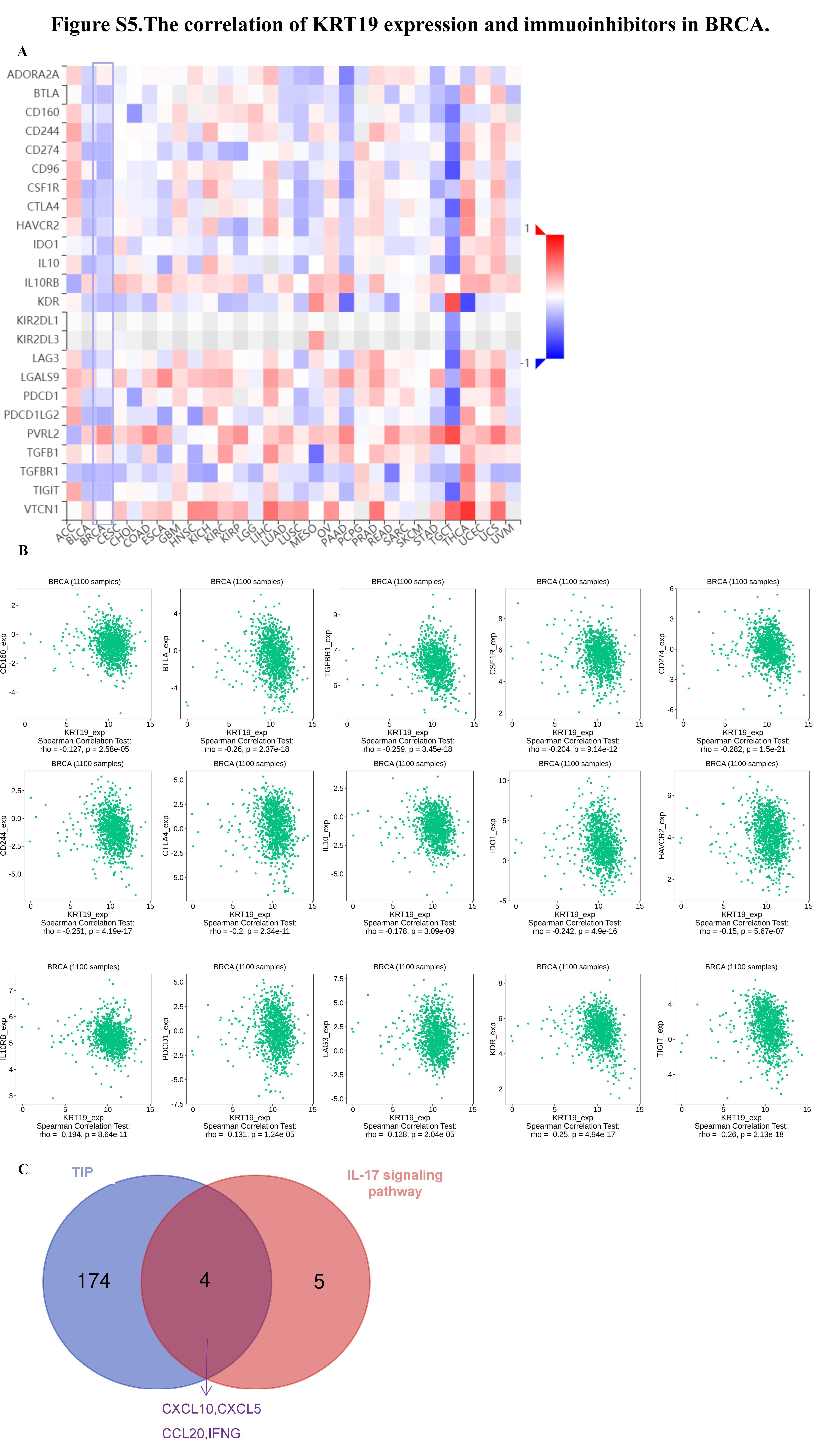

Supplement: Supplementary file 1 [file genes-13-01838-s001.zip › Figure S5.jpg]
